# Management challenges in renal arteriovenous malformation mimicking AVF/pseudoaneurysm without trauma or pathological cause

**DOI:** 10.1016/j.eucr.2025.103107

**Published:** 2025-06-18

**Authors:** Farzad Allameh, Niki Tadayon, Mohammad Amin Tofighi Zavareh, Seyed Mohammad SadrAmeli, Alvand Naserghandi

**Affiliations:** a Men's Health and Reproductive Health Research Center, Shahid Beheshti University of Medical Sciences, Tehran, Iran; b Department of General and Vascular Surgery, Shohada Tajrish Hospital, Shahid Beheshti University of Medical Sciences, Tehran, Iran; c Student Research Committee, Shahid Beheshti University of Medical Sciences, Tehran, Iran

**Keywords:** Renal arteriovenous malformation (AVM), Pseudoaneurysm, Arteriovenous fistula, Embolization, Management

## Abstract

Renal arteriovenous malformations (AVMs) are rare and often pose diagnostic and management challenges, especially in the absence of trauma or underlying pathology. We report a case of a 33-year-old male with a long-standing renal artery aneurysm who presented with flank pain. Imaging revealed a high-flow renal AVM with multiple aneurysms, the largest measuring 30 mm. An attempted embolization was aborted due to the risk of pulmonary embolism. Open surgical repair was performed, involving ligation of the affected arterial branches. This case highlights the complexities in managing non-traumatic renal AVMs and the role of surgery when endovascular treatment is not feasible.

## Highlights

• Renal arteriovenous malformations (RAVMs) are rare vascular anomalies that can mimic AVFs and pseudoaneurysms, making accurate diagnosis essential for proper management.
• This case involved a high-flow renal AVM with associated aneurysms in a patient without trauma or prior interventions, presenting unique diagnostic and therapeutic challenges.
• Endovascular embolization was deemed unsafe due to the high-flow dynamics and risk of pulmonary embolism, necessitating open surgical intervention.
• The successful surgical outcome emphasizes the importance of individualized, multidisciplinary approaches in managing complex renal vascular anomalies.

## 1 Introduction

Renal arteriovenous malformations (RAVMs) are rare vascular anomalies characterized by abnormal, direct connections between arteries and veins, bypassing the normal capillary system. These malformations are congenital.^1^

Proper differentiation among RAVMs, AVFs, and pseudoaneurysms is crucial due to significant differences in clinical management and potential outcomes. Misdiagnosis in this context can lead to inappropriate interventions, increasing the risk of complications such as hypertension, heart failure, and life-threatening hemorrhage, highlighting the importance of accurate identification.^2^

The rarity of RAVMs often results in delayed or overlooked diagnoses, particularly given their variable and sometimes non-specific clinical presentations. Unlike more common renal pathologies, RAVMs may present subtly, with symptoms like hematuria or abdominal pain, potentially misleading clinicians toward alternative diagnoses.^1^^,^^3^

Accurate identification of RAVMs becomes even more essential, as their treatment often involves targeted procedures that differ from the approaches used in AVFs. AVFs, in contrast, are frequently encountered due to their association with trauma or iatrogenic causes and tend to dominate clinical focus, sometimes resulting in diagnostic overshadowing of RAVMs.^1^, ^2^, ^3^

Pseudoaneurysms (PSAs), are notable as a common vascular anomaly resulting from arterial wall disruption, where blood accumulates and creates a false aneurysm cavity.

Accurate and early diagnosis of vascular pathologies is essential to prevent serious complications, particularly in cases prone to rupture, which carries significant morbidity and mortality risks. Allowing for timely intervention and reducing the risk of progression to more severe outcomes.^4^

A thorough understanding of the differential diagnoses is critical to prevent mismanagement.

Overlapping clinical presentations in vascular anomalies continue to pose diagnostic challenges, necessitating ongoing advancements in imaging technologies to enhance diagnostic precision and patient care. Among these imaging modalities, the role of imaging in diagnosing AVMs cannot be overstated. AVMs often exhibit complex vascular architectures involving multiple arterial and venous inputs. Techniques such as ultrasonography (US), magnetic resonance imaging (MRI), and arteriography are essential for accurately visualizing the structural and hemodynamic aspects of these malformations. Arteriography, in particular, remains the gold standard for assessing AVM anatomy and flow dynamics, given its high spatial and temporal resolution, which is crucial for planning effective treatment strategies.^5^, ^6^, ^7^

Given the evolving nature of both imaging and interventional techniques, the management of RAVMs requires a highly individualized approach tailored to the patient's unique vascular anatomy and clinical condition.

This case underscores the need for heightened awareness and precision in diagnosing RAVMs, especially in the absence of trauma or procedural history. Comprehensive imaging and careful consideration of differential diagnoses are essential to ensure appropriate management and to avoid complications related to incorrect treatment pathways. This report highlights the diagnostic and therapeutic complexities encountered in managing RAVMs and contributes to the growing literature on this rare vascular condition.

## 2 Case presentation

A 33-year-old male patient initially discovered an aneurysm of the renal artery approximately 10 years ago during a routine ultrasound screening. Despite the diagnosis, he did not pursue further evaluation or treatment at that time. Recently, however, he began experiencing left flank pain, prompting him to seek medical care. Planned for embolization by the interventional radiology service, the procedure was unsuccessful due to the high flow of the arteriovenous fistula (AVF) and the associated risk of pulmonary embolism. Subsequently, the patient was referred for surgical intervention.

The patient's abdominal aorta CT angiography with 3D reconstruction confirmed a large anomalous renal arteriovenous communication with multiple associated aneurysms, the largest measuring 30 mm in diameter. There was early opacification of the renal vein on the arterial phase, suggesting an AVF. Additional findings included multiple small stones (6 mm, 5 mm, and 7 mm) in the lower and mid-pole of the left kidney. No vascular anomalies were observed in the right kidney, and the abdominal aorta, celiac trunk, superior mesenteric artery (SMA), and inferior mesenteric artery (IMA) appeared normal. Other abdominal organs, including the liver, gallbladder, pancreas, and spleen, were unremarkable, with patent portal and splenic veins and no compressive masses.

During the initial angiographic assessment, a 5F femoral sheath was placed on the right side. Selective renal angiography revealed two branch renal arteries for the left kidney: an upper branch supplying 75 % of the kidney, which was healthy, and a tortuous lower branch supplying the remaining 25 %The lower pole artery in mid portion became aneurysmal and showed a high-flow AVM. Due to proximity to the aorta and the tortuous configuration, the high-flow AVM and potential embolic complications, further endovascular measures were not pursued and surgical intervention was recommended.

**Fig. 1:**
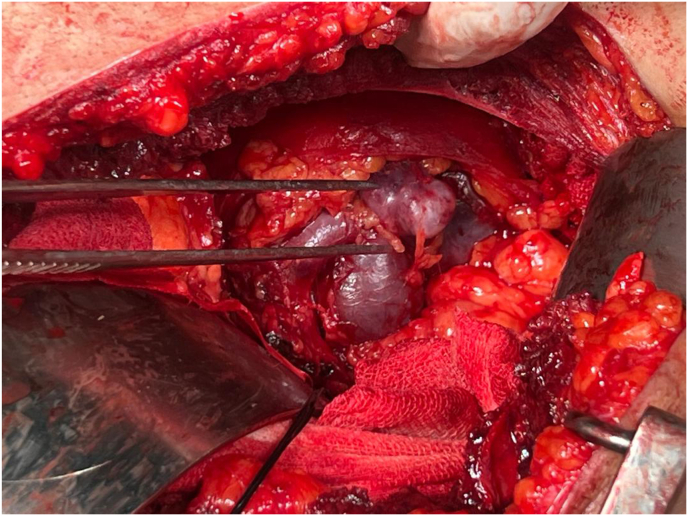
Tortuous configuration.

**Fig. 2:**
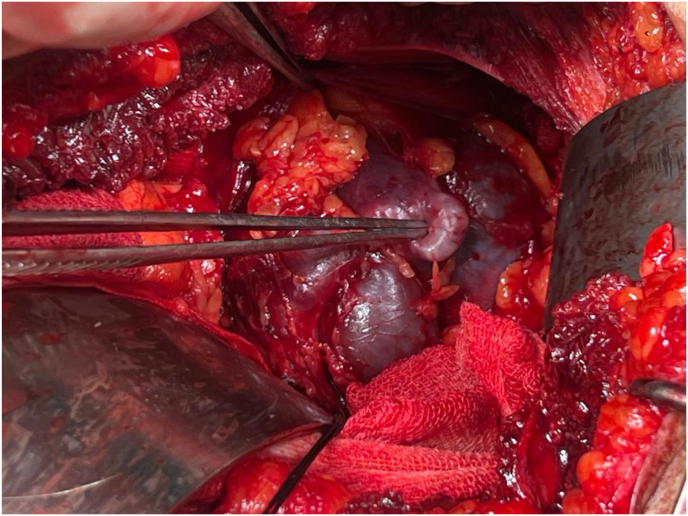
Aneurysmal dilatation.

The patient was scheduled for open surgery. Under general anesthesia, a left subcostal incision provided access to the kidney, Gerota's fascia was opened, and a clear thrill over the left renal aneurysm was palpable. Both the renal artery and vein were clamped using Satinsky clamps. The tortuous lower branch was double ligated. Upon opening the clamps persistent pulsation in the renal vein led to further exploration, revealing an aneurysm at the renal hilum. The aneurysm was opened. Examination revealed two arterial branches superiorly and three inferiorly all opening to aneurysmal sac, they were ligated (Fig. 1, Fig. 2, Video 1). The surgery was completed successfully without intraoperative complications. Postoperatively, the majority of the kidney remained viable, three weeks postoperatively, a contrast-enhanced CT scan demonstrated that more than half of the kidney exhibited contrast uptake on nephrogram phase, with normal contrast excretion into the renal pelvis. No evidence of necrosis was observed in the renal pelvis or ureter. Additionally, a DMSA scan performed three months later confirmed preserved renal function in the remaining renal parenchyma. The patient's renal function remained stable, with no significant change in serum creatinine or estimated glomerular filtration rate (eGFR) at discharge. No postoperative complications such as bleeding, infection, or pulmonary embolism were observed.

Supplementary data related to this article can be found online at https://doi.org/10.1016/j.eucr.2025.103107

The following are the Supplementary data related to this article:

Vid 1: Open surgery.Multimedia component 1

## 3 Discussion

AVMs, which are fast-flow vascular malformations, are complex networks of primitive vessels directly connecting feeding arteries to draining veins with a partial or whole absence of the typical capillary network. Renal AVMs can show a wide range of clinical symptoms, including hypertension, abdominal mass, flank bruit, hematuria, perinephric hematoma, flank pain, and silent presentation.^8^ AVMs mimic a variety of renal pathologies, including hydronephrosis, renal tumors, and cysts, leading to misdiagnosis, which can occasionally result in pointless tests or even have dangerous repercussions.^9^ These complications can cause severe morbidity, especially due to hematuria, which has a differential diagnosis with many diseases.^10^ All these show the insidious manifestations of AVMs and how they can be hard to diagnose correctly and need fast management. Imaging modalities are important in the diagnosis of renal AVMs.^11^ Ultrasound is generally used as a first-line modality because it is non-invasive and provides important information on the diameter of the vessels, flow patterns, and collateral circulation. However, its limitations in complex vascular anomalies necessitate advanced imaging techniques like contrast-enhanced CT angiography or MRI, which allow for detailed visualization of vascular structures and flow dynamics. CT angiography demonstrates characteristic findings of the anomaly and the large aneurysmal structure.^12^ However, despite such advanced imaging, angiography remains the gold standard for a final diagnosis because none can surpass the level of detail in vascular anatomy and shunting dynamics.^13^

This case is particularly notable due to the absence of any identifiable traumatic, iatrogenic, or pathological cause — an uncommon finding in reported renal AVMs, which are typically secondary to prior interventions or trauma. Moreover, the presence of a large, high-flow AVM with associated aneurysmal changes and risk of pulmonary embolism posed a significant management challenge, necessitating open surgical intervention. This contrasts with many reported cases where endovascular treatment alone sufficed. The complexity of the vascular anatomy, combined with the risk factors, required an individualized multidisciplinary approach, underscoring the importance of tailored management strategies in rare vascular anomalies. The imaging findings, including early venous opacification and tortuous vascular structures with a large aneurysm measuring 30 mm, highlighted the intricate vascular anatomy that required careful differentiation from other entities, such as pseudoaneurysms or primary AVFs.

Treatment options for AVMs vary depending on their location, size, and the symptoms they produce. Conventional surgery is often preferred for accessible, smaller, and superficial AVMs, as it eliminates the risk of rupture and bleeding. This minimally invasive procedure involves inserting a catheter to deliver materials that block blood flow to the AVM, reducing its size allowing the rest of treatment to proceed more safely, though it typically does not fully resolve the issue. Radiation therapy, particularly stereotactic radiosurgery like Gamma Knife, is another option. This non-invasive approach uses focused radiation to damage the AVM's blood vessels, leading to gradual shrinkage over time, which is effective for deeper or more complex AVMs.^14^^,^^15^

This case was complicated to manage. The high-flow dynamics of the AVF and the presence of aneurysms, with a significant risk of pulmonary embolism during embolization, make this case exceptional. These risks and the necessity for a definitive treatment guided the decision for surgical intervention. Surgical exploration enabled the ligation of affected branches and repair of the aneurysm by giving direct access to the complex vascular anatomy. This approach was invasive but allowed for controlling hemorrhage, reduced the risk of embolic complications, and provided an opportunity to preserve more healthy renal tissue.

Renal AVMs are rare and each case will bring new information on this topic. Several published cases have demonstrated the diagnostic challenges and variable presentations of renal AVMs. These reports emphasize the utility of advanced imaging techniques, particularly CT angiography and digital subtraction angiography, in differentiating AVMs from other renal pathologies such as malignancies, hydronephrosis, or pseudoaneurysms^16^, ^17^, ^18^, ^19^. Furthermore, minimally invasive treatments such as transarterial embolization have shown excellent success rates with fewer complications, highlighting the importance of individualized treatment planning based on anatomical and hemodynamic characteristics. These cases emphasize the need for thorough evaluation and tailored management in patients with renal symptoms to ensure effective treatment outcomes.

The successful outcome of this case highlights the importance of the multidisciplinary approach. Interventional radiology and urology teams' collaboration was essential in handling the complexities of diagnosis and management. Such integrated models ensure comprehensive care, seamless transitions across various stages of diagnostics and treatment, and better outcomes. Assessment and management protocols for suspected renal vascular anomalies should be established according to the standardized imaging and interdisciplinary consultations in order to enhance care in such rare and complex conditions.

## CRediT authorship contribution statement

**Farzad Allameh:** Writing – review & editing, Supervision, Project administration. **Niki Tadayon:** Writing – review & editing, Supervision, Project administration. **Mohammad Amin Tofighi Zavareh:** Writing – original draft. **Seyed Mohammad SadrAmeli:** Writing – original draft. **Alvand Naserghandi:** Writing – review & editing, Supervision, Project administration.

## Consent

Written informed consent to publish the data and photographs in medical journals was obtained from the patient.

## Funding statement

No financial support was received from any organization or person for this study.

## Declaration of competing interest

All authors confirm that there were no conflicts of interests.
